# Apelin Improves Prognostic Value of HFSS (Heart Failure Survival Score) and MAGGIC (Meta-Analysis Global Group in Chronic Heart Failure) Scales in Ambulatory Patients with End-Stage Heart Failure †

**DOI:** 10.3390/jcm9072300

**Published:** 2020-07-20

**Authors:** Wioletta Szczurek, Mariusz Gąsior, Michał Skrzypek, Bożena Szyguła-Jurkiewicz

**Affiliations:** 1 Silesian Center for Heart Diseases in Zabrze,41-800 Zabrze, Poland; 2 3rd Department of Cardiology, School of Medical Sciences in Zabrze, Medical University of Silesia, 40-055 Katowice, Poland; m.gasior@op.pl (M.G.); bjurkiewicz@sum.edu.pl (B.S.-J.); 3 Department of Biostatistics, School of Public Health in Bytom, Medical University of Silesia, 40-055 Katowice, Poland; mskrzypek@sum.edu.pl

**Keywords:** scales, apelin, heart failure, risk stratification, prognosis

## Abstract

This prospective study aimed to determine the effect of adding apelin to the MAGGIC (Meta-Analysis Global Group In Chronic Heart Failure) and HFSS (Heart Failure Survival Score) scales for predicting one-year mortality in 240 ambulatory patients accepted for heart transplantation (HT) between 2015–2017. The study also investigated whether the combination of N-terminal pro-brain natriuretic peptide (NT-proBNP) with MAGGIC or HFSS improves the ability of these scales to effectively separate one-year survivors from non-survivors on the HT waiting list. The median age of the patients was 58.0 (51.50.0–64.0) years and 212 (88.3%) of them were male. Within a one year follow-up, 75 (31.2%) patients died. The area under the curves (AUC) for baseline parameters was as follows—0.7350 for HFSS, 0.7230 for MAGGIC, 0.7992 for apelin and 0.7028 for NT-proBNP. The HFSS-apelin score generated excellent power to predict the one-year survival, with the AUC of 0.8633 and a high sensitivity and specificity (80% and 78%, respectively). The predictive accuracy of MAGGIC-apelin score was also excellent (AUC: 0.8523, sensitivity of 75%, specificity of 79%). The addition of NT-proBNP to the HFSS model slightly improved the predictive power of this scale (AUC_HFFSS-NT-proBNP_: 0.7665, sensitivity 83%, specificity 60%), while it did not affect the prognostic strength of MAGGIC (AUC_MAGGIC-NT-proBNP_: 0.738, sensitivity 71%, specificity 69%). In conclusion, the addition of apelin to the HFSS and MAGGIC models significantly improved their ability to predict the one-year survival in patients with advanced HF. The MAGGIC-apelin and HFSS-apelin scores provide simple and powerful methods for risk stratification in end-stage HF patients. NT-proBNP slightly improved the prognostic power of HFSS, while it did not affect the predictive power of MAGGIC.

## 1. Introduction

The population of patients with end-stage heart failure (HF) is steadily growing due to a worldwide increase in life expectancy, as well as a more effective pharmacological and interventional treatment of the disease [1,2,3]. As a result, an increasing number of ambulatory patients with end-stage HF are entered on transplant waiting lists, whereas the number of potential heart donors steadily decreased over the past decade [4,5,6]. Furthermore, patients with HF constitute an etiologically and functionally heterogeneous group, which generally makes it difficult to accurately assess the prognosis with the use of any individual parameters [7,8,9]. It seems that prognostic scales that enable a holistic assessment of patient prognosis, are a more reliable tools for assessing the risk of death [5,9,10,11,12,13]. Although there are many risk models with their own set of advantages and disadvantages in end-stage HF, including the Heart Failure Survival Score (HFSS) and Meta-Analysis Global Group in Chronic Heart Failure (MAGGIC) score [11,14], there is still a critical need to improve reliable prognostic tools in ambulatory patients listed for heart transplantation (HT). 

Apelin—an endogenous peptide identified as a ligand for angiotensin-like receptor 1 (APJ)—may play an important role in the pathogenesis of HF by influencing the effects of angiotensin II. Animal and human studies suggest a role for reduced apelin levels in the pathogenesis of HF [15,16]. The serum apelin level decreases with an increasing stage of HF severity and may be an important prognostic factor in HF [17,18,19]. Because of the close relationship between the apelin serum concentrations and left ventricular remodeling as well as regulation of vascular tone we aimed to determine the effect of adding apelin to the MAGGIC and HFSS scores for predicting one-year mortality in ambulatory HF patients listed for HT. In addition, we investigated whether the combination of N-terminal pro-brain natriuretic peptide (NT-proBNP) with MAGGIC or HFSS improved the prognostic strength of these scales in our study population.

## 2. Materials and Methods

### 2.1. Study Population and Data Collection

We prospectively analyzed 287 consecutive ambulatory patients with end-stage HF, who were hospitalized in the Cardiology Department and were put on the HT waiting list between 2015 and 2017. Patients who underwent HT or MCS (mechanical circulatory support) implantation during a one-year follow-up (*n* = 47) were excluded from the study. 

At the time of enrollment to the study, a panel of laboratory tests, an ergospirometric exercise test, echocardiography and right heart catheterization were performed in all patients. In addition, 10 mL of peripheral blood was collected to determine serum apelin concentration. Survival data were collected by telephone contact with patients or their family members or during protocol control visits in our institution. The endpoint was defined as all-cause mortality within 12 months from the date of inclusion to the HT waiting list. The Medical University of Silesia’s local Institutional Review Board approved the study protocol and all patients provided informed consent (specific ethics code—KNW/0022/KB1/88/15). The study was performed in accordance with the ethical standards as laid down in the 1964 Declaration of Helsinki and its later amendments.

### 2.2. Laboratory Measurements

Human apelin was measured by the sandwich enzyme-linked immunosorbent assay (ELISA) with the commercially available kit (Human Apelin ELISA, SunRedBio Technology Co., Ltd., Shanghai, China). The concentration of apelin was expressed as pg/mL. The inter- and intra-assay coefficients of variations (CV) were <12% and <10%. Assay range—0.7 pg/mL→220 pg/mL. The minimum detectable concentration for apelin was 0.658 pg/mL. The complete blood count and hematologic parameters of patients have been analyzed using automated blood cell counters (Sysmex XS1000i and XE2100, Sysmex Corporation, Kobe, Japan). The intra-assay and inter-assay coefficients of variation of the blood samples were 5% and 4.5%, respectively. Hepatic and renal function parameters, as well as cholesterol and albumin plasma concentrations, were determined with a COBAS Integra 800 analyzer (Roche Instrument Center AG, Rotkreuz, Switzerland). The plasma concentration of fibrinogen was measured using the STA Compact analyzer (Roche). A highly sensitive latex-based immunoassay was used to detect the plasma C-reactive protein with the Cobas Integra 70 analyzer (Roche Diagnostics, Ltd.). The C-reactive protein levels were determined with a typical detection limit of 0.0175 mg/dL. The plasma concentration of NT-proBNP was measured with a commercially available kit from Roche Diagnostics (Mannheim, Germany) on an Elecsys 2010 analyzer with the analytical sensitivity of <5 pg/mL.

### 2.3. Analyzed Scales

To calculate the combined HFSS-apelin and MAGGIC-apelin scales, the following formulas were used: 

HFSS was calculated according to the formula described by Aaronson [11]: ([0.0216 × resting heart rhythm] + [−0.0255 × mean arterial blood pressure] + [−0.0464 × LVEF] + [−0.0470 × serum sodium] + [−0.0546 × peak *oxygen consumption*] + [0.6083 × presence (1) or absence (0) of interventricular conduction defect (QRS duration ≥ 0.12 due to any cause)] + [0.6931 × presence (1) or absence (0) of ischemic cardiomyopathy]).

The MAGGIC score [14] was calculated using a calculator available at www.heartfailurerisk.org. The scale includes 13 parameters: age, gender, body mass index, systolic blood pressure, creatinine concentration, presence or absence of diabetes mellitus and chronic obstructive pulmonary disease, HF, diagnosed within the last 18 months, New York Heart Association (NYHA) class, left ventricular ejection fraction (LVEF), current smoking status, use of β-blockers and angiotensin converting enzyme inhibitors (ACEIs) or angiotensin receptor blockers (ARBs).

The scores for HFSS and apelin, as well as (separately) for MAGGIC and apelin were included in the Cox regression model as a continuous variables and each variable was multiplied by its corresponding β-coefficient. The same calculations were performed for the combination of MAGGIC and NT-proBNP, as well as HFSS and NT-proBNP. The final scores for new scales were calculated based on the following formulas:

MAGGIC-apelin = 0.15656 × MAGGIC−0.05987 × APELIN

HFSS-apelin = −1.13869 × HFSS −0.06516 × APELIN

MAGGIC-NT-proBNP = 0.16236 × MAGGIC + 0.0000483 × NT-proBNP

HFSS-NT-proBNP = −0.99365 × HFSS + 0.000047 × NT-proBNP

The raw score for HFSS-apelin was multiplied by (−1), to achieve a positive value and facilitate the interpretation of results.

### 2.4. Statistical Analysis

The statistical analysis was performed using SAS software, version 9.4 (SAS Institute Inc., Cary, NC, USA). Continuous variables are expressed as mean and standard deviation for normally distributed variables or as median and upper and lower quartiles for non-normal distributions. Categorical variables are described as counts and percentages. Differences between the study groups were assessed using Student’s *t* test, the Mann-Whitney test or χ^2^ test. To evaluate the prognostic utility of the new models for one-year mortality, receiver operating characteristic (ROC) curves analysis was performed. The prognostic strength of the new models was evaluated by calculating each area under the ROC curve (AUCs), sensitivity, specificity, the negative predictive value (NPV), the positive predictive value (PPV), the negative likelihood ratio (LR-), the positive likelihood ratio (LR+) and accuracy. The Youden Index was calculated from the ROC curve analysis to establish the optimized cut-off point. The ROC curves were quantitatively compared using the DeLong test, while the differences between AUC values were tested using the Hanley and McNeil method. Kaplan-Meier curves with the log-rank test were performed to compare mortality rates in patients dichotomized according to the cut-off values from the ROC curves for the HFSS-apelin and MAGGIC-apelin score. A *p*-value <0.05 was considered as statistically significant.

## 3. Results

The final study group consisted of 240 patients with end-stage HF awaiting HT. All participants were classified in New York Heart Association (NYHA) functional classes III and IV (86.7% and 13.3%, respectively) and profiles 4 to 6 according to the Interagency Registry for Mechanically Assisted Circulatory Support (INTERMACS) classification. Within a one-year follow-up, 75 (31.2%) patients died. The values of the scales components at the time of inclusion to the study are presented in Table 1. Complementary characteristics of the study population are shown in Table 2.

**Figure 1:**
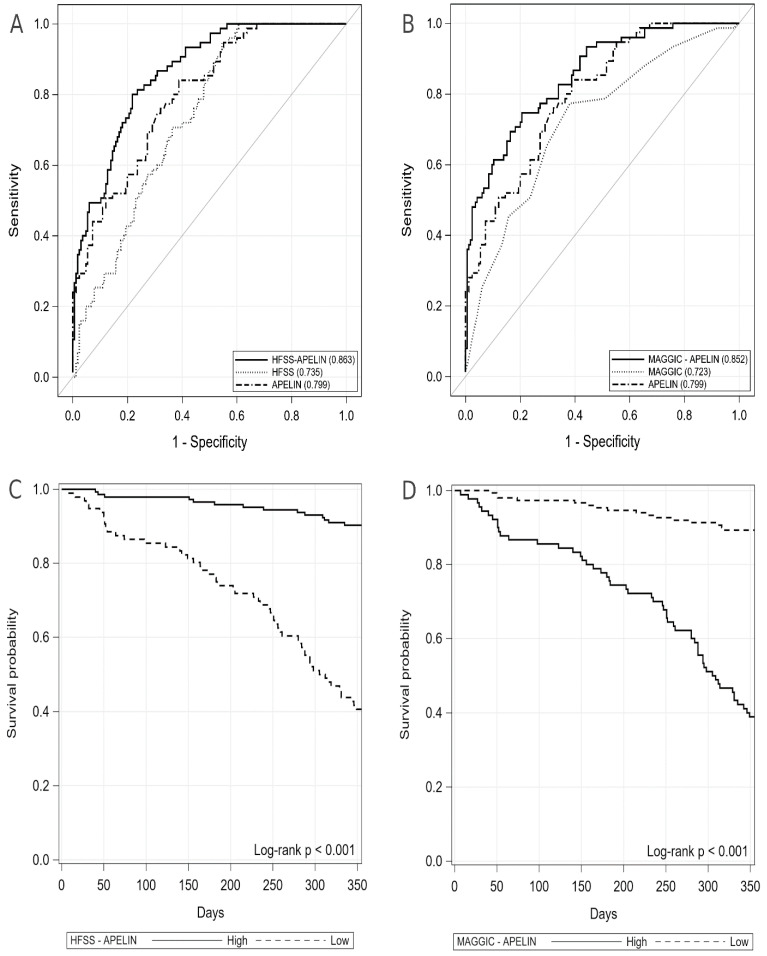
The ROC curves and Kaplan–Meier survival curves for HFSS-apelin and MAGGIC-apelin scores. **A**—The ROC curve for HFSS-apelin and its components; **B**—The ROC curve for MAGGIC-apelin and its components, **C**—The Kaplan-Meier curve for HFSS-apelin, **D**—The Kaplan-Meier curve for MAGGIC-apelin. Abbreviations: HFSS, Heart Failure Survival Score; MAGGIC, Meta-Analysis Global Group in Chronic Heart Failure.

The ROC curves and Kaplan–Meier survival curves for the HFSS-apelin and MAGGIC-apelin scores are shown in Figure 1A–D. The AUCs for the new scores generated an excellent power to predict one-year mortality (AUC_HFSS-apelin_ = 0.8633 [95% CI: 0.8176–0.9090]; AUC_MAGGIC-apelin_ = 0.8523 [95% CI: 0.8016–0.9029]), as well as high sensitivity and specificity (HFSS-apelin: 80% and 78%; MAGGIC-apelin: 89% and 72%; respectively). A summary of the ROC analysis for new scales and their components are presented in Table 3.

An improvement in the AUC and *p* values for one-year mortality was observed in both the combination of apelin with the HFSS scale and the MAGGIC scale, relative to those individual components. The difference between the calculated AUCs for HFSS-apelin and apelin amounted to 0.064 (95% CI: 0.025–0.104), while that the difference between the AUCs for HFSS-apelin and HFSS was 0.128 (95% CI: 0.074–0.183), both being statistically significant (*p* = 0.0014 and *p* < 0.001, respectively). Similarly, the differences between the AUCs for the MAGGIC-apelin and apelin [0.053 (95% CI: 0.015–0.091), *p* = 0.006], as well as between the MAGGIC-apelin and MAGGIC levels [0.129 (95% CI: 0.074–0.184), *p* < 0.001] were statistically significant. When NT-proBNP was added to the HFSS scale, an improvement in the prognostic value was also obtained (the difference between the AUCs for HFSS-NTproBNP and HFSS was 0.0315 [0.0053–0.0578], *p* = 0.0186), although an increase in the AUC for HFSS-NT-proBNP was significantly lower compared with the combination of HFSS and apelin (*p* < 0.001). On the other hand, the addition of NT-proBNP to MAGGIC did not improve its prognostic power (the difference between the AUCs for MAGGIC-NTproBNP and MAGGIC amounted to 0.0152 ([0.0068–0.0372], *p* = 0.1744), while the difference between the calculated AUCs for MAGGIC-NT-proBNP and NT-proBNP was 0.0355 ([0.0365–0.1074], *p* = 0.3340).

According to Kaplan-Meier curves (Figure 1), a lower HFSS-apelin score (≤10.84) was associated with a significantly inferior one-year survival compared to a higher HFSS-apelin score (>10.84) [one-year survival: 37.5 % versus 89.6%; log rank *p* < 0.001]. In turn, a higher MAGGIC-apelin score (> 1.885) was associated with a significantly inferior prognosis compared to a lower MAGGIC-apelin score (≤1.885) [one-year survival: 37.8 % versus 87.3%; log rank *p* < 0.001].

## 4. Discussion

The present study is the first one to demonstrate that the modified MAGGIC-apelin and HFSS-apelin scores provide simple and powerful methods for risk stratification of one-year mortality in ambulatory patients awaiting HT. The MAGGIC-apelin and HFSS-apelin models have an excellent power, as well as good sensitivity and specificity, allowing for an effective separation of one-year survivors from non-survivors on the HT waiting list. 

Despite a number of tests performed in the course of the qualification process for HT, there is still a great need to find additional prognostic tools for evaluating the prognosis on HT waiting list in patients with end-stage HF. Original HFSS and MAGGIC scores were validated and widely used predictive models in patients with chronic HF [11,14]. The HFSS model was developed in the 1990s by Aaronson from a single center cohort of 268 ambulatory patients referred for HT evaluation and has been prospectively validated in a similar group of 199 patients [11]. Over the years, the HFSS scale has been validated in many independent external cohorts, with a discriminative power ranging from 0.56 to 0.81 [3,7,11,20]. The MAGGIC scale was originally developed in 2012 by Pocock et al. based on the data of 39,372 patients with HF [14] and its acceptable discriminatory power (0.67–0.77) has been confirmed in several external analyzes. However, the previous studies have not analyzed the predictive ability of these scores to assess a one-year mortality rate after the inclusion of apelin level into these models. Apelin is a relatively new marker in chronic HF. It is an endogenous peptide that acts through the APJ, which shows similarities with the angiotensin II—angiotensin II type 1 (AT 1) receptor [17]. Clinical and animal studies indicate that apelin plays an important role in cardiovascular homeostasis processes by being involved in body fluid regulation, endocrine stress response, cardiac contractility, angiogenesis and inflammatory processes, as well as in vasodilator actions [16,17,18,19]. In our study, apelin level showed a good prognostic power and the addition of this marker to the HFSS and MAGGIC models significantly improved their prognostic capability to assess one-year mortality. The differences between the AUCs for the new scales and their components were significant, which indicates that HFSS-apelin and MAGGIC-apelin are significantly better predictors for one-year mortality in ambulatory HF patients than the individual components. Our results can imply that patients who present higher risk of death according to the HFSS-apelin and MAGGIC-apelin scores should be considered as urgent candidates for HT or MCS implantation.

In our study, we also investigated the usefulness of combining NT-proBNP with MAGGIC and separately with HFSS in the assessment of one-year mortality in patients with end-stage HF. The addition of NT-proBNP to the MAGGIC scale did not improve its predictive ability to effectively separate one-year survivors from non-survivors on the HT waiting list. However, NT-proBNP slightly improved the prognostic power of the HFSS score to predict one-year mortality in the analyzed group of patients. Natriuretic peptides play an important regulatory role in responding to the increase in ventricular volume by opposing vasoconstriction, sodium retention and antidiuretic effects of the activated renin-angiotensin-aldosterone system [12,21]. In clinical practise, NT-proBNP is commonly used to aid the diagnosis of HF, assess the effect of therapy and predict the outcomes at different stages of HF [1,22,23,24]. However, not only is NT-proBNP specific for HF but its concentration increases in many clinical conditions, such as kidney dysfunction, atrial fibrillation, diabetes mellitus, cardiac arrhythmias, pulmonary hypertension, older age or obesity [1,21,23]. These conditions may limit the value of NT-proBNP to predict survival in patients with end-stage HF. Furthermore, as our previous study has shown [12], the relatively limited prognostic power of NT-proBNP may result from the fact that our population included a selected optimally treated group of stable patients with end-stage HF. Optimal neurohormonal suppression with maximal HF therapy may also limit the prognostic performance of this marker and contribute to the lack of significant improvement of prognostic strength in the combination of NT-proBNP with HFSS and MAGGIC, as compared to adding apelin to these scales.

It should be emphasized that this study has several limitations. First, this is a single-center study involving a relatively small group of patients. In addition, the study population is drawn from a selected group of patients with HF and so larger, multicenter and prospective studies are required to further confirm the usefulness of these scores in clinical practice. Furthermore, there is no independent validation cohort that would support the prognostic value of apelin in this group of patients. It is likely that if an independent validation cohort had been used, the AUC for apelin would have been lower. There is also a need to determine whether pre-transplant apelin levels affect long-term survival after heart transplantation.

This study may bear clinical importance, as it provides simple and effective methods for the risk stratification of one-year mortality in ambulatory patients with end-stage HF awaiting HT. To the best of our knowledge, this is the first study to demonstrate that the addition of apelin to the HFSS and MAGGIC models significantly improves their prognostic ability to predict the one-year mortality in patients awaiting HT. It seems that the modified HFSS-apelin and MAGGIC-apelin scores may support the risk stratification of one-year mortality in end-stage HF patients and facilitate the selection of candidates for HT. The addition of NT-proBNP to the HFSS or MAGGIC scores does not significantly improve the ability of these scales to predict one-year mortality, as compared with the combination of HFSS with apelin and MAGGIC with apelin.

## Figures and Tables

**Table 1:** Components of the evaluated scales.

	All Included (*n* = 240) ^#^	Survivors (*n* = 165)	Non-Survivors (*n* = 75)	*p* *
**HFSS components**				
Ischemic etiology of HF, %	118 (49.2)	78 (47.3)	40 (53.3)	0.3840
Rest HR, beats per min	72.00 (65.00–79.00)	70.00 (65.00–78.00)	75.00 (67.00–80.00)	0.3917
Rest mBP, mmHg	74.81 (9.36)	75.68 (9.13)	72.91 (9.64)	0.0334 *
Sodium, mmol/L	139 (137–141)	140 (138–141)	138 (135–139)	<0.0001 *
VO_2_ max, mL/kg/min	11.20 (10.30–12.20)	11.30 (10.40–12.30)	10.90 (9.70–12.20)	0.1544
Presence of IVCD, *%*	105 (43.8)	70 (42.4)	35 (46.7)	0.5392
**MAGGIC components**				
Age, years	58.0 (51.50.0–64.0)	57.00 (50.00–63.00)	60.00 (54.00–65.00)	0.0517
Male, %	212 (88.3)	142 (86.1)	70 (93.3)	0.1038
NYHA III, %	208 (86.7)	149 (90.3)	59 (78.7)	0.014 *
NYHA IV, %	32 (13.3)	16 (9.7)	16 (21.3)	0.014 *
HF diagnosed within the last 18 months, %	12 (5.0)	7 (4.2)	5 (6.7)	0.4245
Current smoker, %	37 (15.4)	25 (15.2)	12 (16)	0.8660
Type 2 diabetes, %	96 (40)	62 (37.6)	34 (45.3)	0.2555
COPD, %	27 (11.3)	18 (10.9)	9 (12)	0.8042
BMI, kg/m^2^	27.02 (24.13–30.47)	27.36 (24.69–31.18)	25.66 (22.63–29.54)	0.014 *
Resting SBP, mmHg	100 (92–110)	102 (97.–114)	100 (90–103)	<0.0001 *
Creatinine, µmol/L	107 (94.5–125.5)	104 (88.6–110)	127 (106–147)	<0.0001 *
B-blockers, %	238 (99.2)	163 (98.8)	75 (100)	0.8725
ACEI/ARB, %	234 (97.5)	160 (97)	74 (98.7)	0.4351
**Common to HFSS and MAGGIC**				
LVEF, %	17.00 (15.00–20.00)	18.00 (15.00–20.00)	15.00 (13.00–18.00)	0.0003 *
**SCORES**				
HFSS	7.54 (7.16–8.00)	7.73 (7.33–8.19)	7.29 (6.92–7.62)	<0.0001 *
MAGGIC	25.00 (22.00–27.50)	24.00 (22.00–26.00)	27.00 (25.00–30.00)	<0.0001 *
Apelin, pg/mL	37.88 (29.65–52.30)	42.01 (34.43–64.87)	29.27 (21.04–36.55)	<0.0001 *
NT-proBNP, pg/mL	2854.5 (1657–6189)	2026 (1553–4674)	5093 (2437–7856)	<0.0001 *
HFSS-apelin	11.20 (10.35–12.24)	11.62 (10.99–13.28)	10.26 (9.48–10.77)	<0.0001 *
MAGGIC-apelin	1.55 (0.49–2.15)	1.09 (0.10–1.77)	2.51 (1.75–3.20)	<0.0001 *
HFSS-NT-proBNP	7.33 (6.82–7.75)	7.48 (7.10–7.97)	6.87 (6.63–7.32)	<0.0001 *
MAGGIC-NT-proBNP	4.22 (3.80–4.79)	3.99 (3.65–4.44)	4.65 (4.15–5.17)	<0.0001 *

Abbreviations: ACEI, angiotensin-converting-enzyme inhibitor; ARB, Angiotensin II receptor blocker; BMI, body mass index; COPD, chronic obstructive pulmonary disease; HF, heart failure; HFSS, Heart Failure Survival Score; HR, heart rhythm; IVCD, intraventricular conduction defect; LVEF, left ventricular ejection fraction; MAGGIC, Meta-Analysis Global Group in Chronic Heart Failure; mBP, mean blood pressure; NYHA, New York Heart Association; SBP, systolic blood pressure; VO_2_, oxygen consumption. ^#^ Data are presented as medians (25th–75th percentile), means (standard deviation) or numbers (percentages) of patients. * statistical significance *p* < 0.05.

**Table 2:** Complementary characteristics of the study populations.

	All Included # (*n* = 240)	Survivors (*n* = 165)	Non-Survivors (*n* = 75)	*p*
**Comorbidities**				
Hypertension, %	140 (58.3)	88 (53.3)	52 (69.3)	0.0198 *
Persistent atrial fibrillation, %	116 (48.3)	84 (50.9)	32 (42.7)	0.2363
Hypercholesterolemia, %	155 (64.6) 0 0	105 (63.6)	50 (66.7)	0.6491
Pulmonary hypertension, %	65 (27.1)	42 (25.5%)	23 (30.7%)	0.3997
**Laboratory parameters**				
Leukocytes, ×10^9^/L	7.68 (6.08–8.89)	7.33 (6.06–8.68)	8.05 (6.56–9.14)	0.0856
Haemoglobin, mmol/L	8.83 (0.97)	8.81 (0.94)	8.88 (1.05)	0.6334
Platelets, ×10^9^/L	186.00 (158.50–232.00)	185.00 (158.00–232.00)	191.00 (159.00–235.00)	0.8326
Total bilirubin, µmol/L	15.85 (11.65–20.95)	15.30 (11.50–19.60)	18.50 (11.70–23.00)	0.0212 *
Albumin, g/L	43.00 (41.00–46.00)	44.00 (42.00–46.00)	42.00 (38.00–44.00)	<0.0001 *
Uric acid, µmol/L	424.50 (360.00–512.50)	421.00 (353.00–506.00)	447.00 (366.00–515.00)	0.3751
Urea, µmol/L	8.25 (5.90–13.15)	8.00 (5.70–10.30)	10.60 (6.60–17.80)	0.0014 *
Fibrinogen, mg/dl	379.00 (312.50–442.00)	366.00 (309.00–432.00)	396.00 (324.00–459.00)	0.0471 *
AST, U/L	26.00 (20.00–31.50)	26.00 (20.00–32.00)	25.00 (19.00–31.00)	0.7384
ALT, U/L	22.00 (15.00–33.00)	22.00 (17.00–33.00)	20.00 (14.00–33.00)	0.1711
ALP, U/L	77.00 (62.00–100.00)	75.00 (61.00–97.00)	86.00 (64.00–104.00)	0.0567
GGTP, U/L	69.00 (34.00–125.00)	69.00 (32.00–125.00)	69.00 (40.00–123.00)	0.3524
Cholesterol, mmol/L	4.01 (3.28–4.79)	4.03 (3.36–4.75)	3.83 (3.14–4.84)	0.3442
LDL, mmol/L	2.13 (1.64–2.71)	2.14 (1.66–2.79)	2.10 (1.62–2.67)	0.6594
hs-CRP, mg/L	4.12 (1.93–6.88)	3.40 (1.68–5.43)	6.74 (2.79–9.33)	<0.0001 *
ESR, mm/h	14.00 (8.00–21.00)	11.00 (7.00–19.00)	19.00 (12.00–25.00)	<0.0001 *
Glucose, mmol/L	5.69 (0.66)	5.67 (0.66)	5.71 (0.66)	0.6962
HBA1c, %	5.70 (5.35–6.30)	5.80 (5.40–6.30)	5.60 (5.30–6.20)	0.1424
**Hemodynamic parameters**				
PAPm, mmHg	25.00 (19.00–32.00)	25.00 (19.00–31.00)	25.00 (19.00–35.00)	0.5449
PAWPm, mmHg	17.00 (11.50–21.00)	17.00 (12.00–20.00)	17.00 (10.00–23.00)	0.496
PVR, Woods units	1.86 (1.47–2.33)	1.80 (1.46–2.28)	2.00 (1.52–2.35)	0.2938
Cl, L/min/m2	1.93 (1.78–2.01)	1.93 (1.80–2.00)	1.94 (1.77–2.01)	0.7835
**Echocardiographic parameters**				
LA, mm	52.00 (47.00–57.50)	51.00 (47.00–58.00)	54.00 (48.00–57.00)	0.3655
RVEDd, mm	39.00 (35.00–40.00)	38.00 (34.00–40.00)	39.00 (37.00–42.00)	0.0034 *
LVEDd, mm	70.00 (63.50–80.00)	70.00 (63.00–79.00)	71.00 (64.00–81.00)	0.3313
**Cardiac medication**				
B-blockers, %	238 (99.2)	163 (98.8)	75 (100)	0.8725
Loop diuretics, %	238 (99.2)	165 (100)	75 (100)	
MRA, %	240 (100)	165 (100)	75 (100)	
Digoxin, %	70 (29.2)	46 (27.9)	24 (32)	0.5150
Amiodarone, %	50 (20.8)	39 (23.6)	11 (14.7)	0.1127
Statin, %	179 (74.6)	122 (73.9)	57 (76)	0.7340
Coumarin derivatives, %	141 (58.8)	98 (59.4)	43 (57.3)	0.7637
Acetylsalicylic acid, %	91 (37.9)	61 (37)	30 (40)	0.6538
Sildenafil, %	65 (27.1)	42 (25.5)	23 (30.7)	0.3997
ICD/CRT-D, %	240 (100)	165 (100)	75 (100)	
Allopurinol, %	169 (70.4)	121 (73.3)	48 (64)	0.1420

Abbreviations: ALP, alkaline phosphatase; ALT, alanine aminotransferase; AST, aspartate aminotransferase; CI, cardiac index; CRT-D, cardiac resynchronization therapy with defibrillator; ESR, erythrocyte sedimentation rate; GGTP gamma-glutamyl transpeptidase; HBA1c, haemoglobin A1c; hs-CRP, high-sensitivity C-reactive protein; ICD, implantable cardioverter-defibrillator; LA, left atrium; LVEDd, left ventricular end-diastolic diameter; MRA, mineralocorticoid receptor antagonists; NT-proBNP, N-terminal prohormone of brain natriuretic peptide; PAPm, mean pulmonary artery pressure; PAWPm, mean pulmonary capillary wedge pressure; PVR, pulmonary vascular resistance; RVEDd, right ventricular end-diastolic diameter; VO_2_, oxygen consumption. * statistical significance *p* < 0.05. # Data are presented as medians (25th–75th percentile), means (standard deviation) or numbers (percentages) of patients

**Table 3:** A summary of the receiver operating characteristic (ROC) curves analysis for new scales and their components.

	AUC [±95% CI]	Cut-off	Sensitivity [±95% CI]	Specificity [±95% CI]	PPV [±95% CI]	NPV [±95% CI]	LR+ [±95% CI]	LR− [±95% CI]	Accuracy
HFSS	0.7350 [0.6730–0.7971]	≤7.8	0.95 [0.87–0.99]	0.45 [0.38–0.54]	0.44 [0.36–0.52]	0.95 [0.88–0.99]	1.74 [1.47–1.99]	0.12 [0.003–0.23]	0.61 [0.54–0.67]
MAGGIC	0.7230 [0.6538–0.7923]	≥25	0.77 [0.66–0.86]	0.62 [0.54–0.69]	0.48 [0.39–0.57]	0.86 [0.78–0.92]	2.03 [1.56–2.49]	0.37 [0.21–0.53]	0.67 [0.60–0.73]
Apelin	0.7992 [0.7421–0.8562]	≤39.66	0.84 [0.74–0.91]	0.61 [0.53–0.69]	0.50 [0.41–0.59]	0.89 [0.82–0.94]	2.17 [1.70–2.64]	0.26 [0.12–0.40]	0.68 [0.62–0.74]
HFSS-apelin	0.8633 [0.8176–0.9090]	≤10.84	0.80 [0.69–0.88]	0.78 [0.71–0.84]	0.63 [0.52–0.72]	0.90 [0.83–0.94]	3.67 [2.52–4.81]	0.26 [0.14–0.37]	0.78 [0.73–0.84]
MAGGIC-apelin	0.8523 [0.8016–0.9029]	≥1.885	0.75 [0.63–0.84]	0.79 [0.72–0.84]	0.62 [0.52–0.72]	0.87 [0.81–0.92]	3.62 [2.43–4.82]	0.32 [0.19–0.45]	0.78 [0.72–0.83]
NT-proBNP	0.7028 [0.6338–0.7718]	≥2138	0.81 [0.71–0.89]	0.53 [0.45–0.61]	0.44 [0.35–0.53	0.86 [0.78–0.92]	1.72 [1.38–2.06]	0.35 [0.18–0.53]	0.62 [0.55–0.68]
HFSS-NT-proBNP	0.7665 [0.7076–0.8255]	≤7.371	0.83 [0.71–0.89]	0.60 [0.52–0.68]	0.48 [0.39–0.57]	0.88 [0.80–0.93]	2.03 [1.59–2.48]	0.31 [0.16–0.46]	0.67 [0.60–0.73]
MAGGIC-NT-proBNP	0.7380 [0.6705–0.8061]	≥ 4.3	0.71 [0.59–0.81]	0.69 [0.61–0.76]	0.51 [0.41–0.61]	0.84 [0.77–0.90]	2.29 [1.66–2.91]	0.43 [0.27–0.58]	0.70 [0.63–0.75]

Abbreviations: AUC, area under the curve; CI, confidence interval; HFSS, Heart Failure Survival Score; LR−, negative likelihood ratio; LR+, positive likelihood ratio; MAGGIC, Meta-Analysis Global Group in Chronic Heart Failure, NPV, negative predictive value; NT-proBNP, N terminal prohormone of brain natriuretic peptide; PPV, positive predictive value.
